# Antimicrobial Properties of Food Nanopackaging: A New Focus on Foodborne Pathogens

**DOI:** 10.3389/fmicb.2021.690706

**Published:** 2021-07-12

**Authors:** Amir Ali Anvar, Hamed Ahari, Maryam Ataee

**Affiliations:** ^1^Department of Food Hygiene, Science and Research Branch, Islamic Azad University, Tehran, Iran; ^2^Department of Food Science and Technology, Science and Research Branch, Islamic Azad University, Tehran, Iran

**Keywords:** food packaging, nanoparticles, foodborne pathogens, antimicrobial, COVID-19

## Abstract

Food products contaminated by foodborne pathogens (bacteria, parasites, and viruses) cause foodborne diseases. Today, great efforts are being allocated to the development of novel and effective agents against food pathogenic microorganisms. These efforts even might have a possible future effect in coronavirus disease 2019 (COVID-19) pandemic. Nanotechnology introduces a novel food packaging technology that creates and uses nanomaterials with novel physiochemical and antimicrobial properties. It could utilize preservatives and antimicrobials to extend the food shelf life within the package. Utilizing the antimicrobial nanomaterials into food packaging compounds typically involves incorporation of antimicrobial inorganic nanoparticles such as metals [Silver (Ag), Copper (Cu), Gold (Au)], and metal oxides [Titanium dioxide (TiO_2_), Silicon oxide (SiO_2_), Zinc oxide (ZnO)]. Alternatively, intelligent food packaging has been explored for recognition of spoilage and pathogenic microorganisms. This review paper focused on antimicrobial aspects of nanopackaging and presented an overview of antibacterial properties of inorganic nanoparticles. This article also provides information on food safety during COVID-19 pandemic.

## Introduction

Foods or beverages contaminated by foodborne pathogens (bacteria, parasites, and viruses) cause foodborne diseases that are generally classified into foodborne infection and foodborne intoxication. Foodborne infection occurs when a pathogen is ingested with food and establishes and multiplies itself in the human host. Foodborne intoxication occurs when a toxigenic pathogen establishes itself in a food and produces a toxin, which is then ingested by the human host (Bintsis, 2017). World Health Organization (WHO) reports also indicate foodborne diarrheal diseases cause 550 million cases and 230,000 deaths worldwide a year (WHO, 2015). The overall rate of foodborne diseases outbreak leads to a great concern about the infectious food products; therefore great efforts are being allocated to the development of novel and effective agents against food pathogenic microorganisms. These efforts might have a possible future effect in more critical conditions, such as pandemic diseases like influenza viruses, Severe Acute Respiratory Syndrome (SARS), and its newer form, SARS-CoV-2 (corona virus disease 2019, COVID-19).

When considering foods safety, the original source of the food, the microbiological quality of the raw food, the microbiological quality of the processed food, and subsequently the packaging, storage, and distribution are all important. Packaging alone has become a separate area for extensive research into the prevention of foodborne illnesses. Food packaging is universally used to preserve the food quality and to extend the shelf life. Proper packaging could protect food products from microbial damage or from any other type of environmental contamination while a poor quality food packaging increases food waste and foodborne illnesses. Traditional food packages are passive barriers which can only delay the adverse effects of environmental contamination (Brody et al., 2008). In fact the key safety objective for traditional packaging materials is to be inert as possible in contact with food (Biji et al., 2015).

Today, the development of non-conventional packaging is becoming a key research field. The next generation of food packaging can play an important role in reducing the risk of pathogen contamination and extending the shelf-life of foods. Application of nanotechnology presents novel opportunities for exploring the bactericidal effect of nanomaterials with a marked bioactivity (Singh et al., 2017). Nanotechnology is the study and use of matter and structures sized from 1 to 100 nm (the word “nano” means 10^–9^ or one billionth of something) (Sujithra and Manikkandan, 2019).

Nanotechnology introduce a novel food packaging technology for the food industry (Dasgupta et al., 2015). The term nanopackaging comes from this combination of nanotechnology and food packaging shows a direct application of nanotechnology in food science. Nano-sciences create and use nanomaterials with novel physiochemical properties that offer many new opportunities for food industries (Gupta et al., 2016; Singh et al., 2017). More potent food coloring, flavoring, nutritional additives, and antibacterial ingredients for food packaging are the new opportunities for food industries.

Utilizing the antimicrobial nanomaterials into food packaging compounds typically involves incorporation of antimicrobial inorganic nanoparticles (NPs) (Rhim and Ng, 2007). Materials in the nanoscale range with a higher surface-to-volume ratio can attach more copies of microorganism, which confers greater efficiency (Luo and Stutzenberger, 2008). The antimicrobial nanomaterials are especially interesting because of their barrier properties and desirable structural integrity leads to decrease the spoilage and pathogenic microorganism growth (Rhim and Ng, 2007). In antimicrobial films, nanomaterials can be employed as growth inhibitors, killing agents, or even antibiotic carriers. Several studies show the biological effectiveness of inorganic NPs with a strong antibacterial activity in low concentrations (Jafarzadeh et al., 2020). The typical inorganic NPs used for food packaging are silver nanoparticles (AgNPs) that are popular for its excellent toxicity to many microorganisms, with low volatility and high-temperature stability (Shahbazzadeh et al., 2009; Ahari et al., 2020; Kavakebi et al., 2021). The most common method for AgNPs preparation is chemical reduction. The reduction of silver ions in aqueous solution leads to the formation of silver atoms and aggregation into colloidal clusters. These clusters form the Ag particles with nanometer dimensions. Smaller AgNPs have larger surface area to interact with microbial cells that results in better bactericidal efficiency (Azeredo, 2009). Other antimicrobial NPs which have been applied in food packaging are titanium dioxide NPs (TiO_2_NPs). TiO_2_NPs have a photocatalytic activity that creates the polyunsaturated phospholipids peroxidation in the microbial cell membranes. This property has been applied to inactivate several foodborne pathogens (Chorianopoulos et al., 2011). Combining TiO_2_ with metal increases its photocatalytic bacterial inactivation. The combination of TiO_2_/AgNPs has been used in several studies (Barani et al., 2018; Lotfi et al., 2019). In addition to AgNPs and TiO_2_NPs, there are a wide range of nanomaterials introduced to the food industries. Due to the fundamental differences in structural and physicochemical properties, each nanomaterial has different applications in the food nanopackaging. Many informative studies have addressed the practical applications of various nanomaterials in the food packaging industry. However, the number of studies focusing on the application of nanopackaging technology in food microbiology is limited. Despite the antimicrobial effectiveness aspects of nanopackaging, it is important to stress the nanomaterial interactions with food ingredients and possible alterations of food quality ranged from sensory features to safety aspects (Ahari and Lahijani, 2021). This largely depends on the nanomaterial doses and concentrations used in fabrication of nanopackages. Optimizing the right dose and concentration that leads to controlled release of nanoparticle can preserve antimicrobial properties without any alteration in the food quality and safety.

The goals of this study are to focus on antimicrobial aspects of nanopackaging. Here, we present an overview of antibacterial properties of inorganic NPs and highlight their specific effectiveness. Finally, the toxicity of inorganic NPs and their possible danger to human health are discussed.

## Foodborne Pathogens

Foodborne pathogenic microorganisms are a branch of food microbes that may not alter the appearance, taste, and quality of products, but they can contaminate foods and cause foodborne illnesses. Therefore, the food safety could not be assessed based on product appearance alone. According to the Centers for Disease Control and Prevention (CDC), every year, 48 million people get sick in the United States from a foodborne illness, 128,000 are hospitalized, and 3,000 die. Table 1 presents the list of foodborne pathogens involved in foodborne diseases.

**TABLE 1:** Types of food pathogens.

**Microorganism**		
**Foodborne bacteria**	**Foodborne parasites**	**Foodborne viruses**
*Aeromonas hydrophila*	*Cryptosporidium parvum*	Astrovirus
*Bacillus anthracis*	*Cyclospora cayetanensis*	Avian influenza
*Bacillus cereus*	*Cystoisospora belli*	Hepatitis A virus
*Brucella* sp.	*Entamoeba histolytica*	Hepatitis E virus
*Campylobacter* sp.	*Giardia intestinalis*	Norovirus
*Clostridium botulinum*	*Helminths*	Rotavirus
*Clostridium perfringens*	*Taenia solium*/*saginata*	
*Cronobacter sakazakii*	*Toxoplasma gondii*	
*Escherichia coli*	*Trematodes*	
*Escherichia coli* O157:H7	*Trichinella spiralis*	
*Listeria monocytogenes*		
*Mycobacterium paratuberculosis*		
*Salmonella* sp.		
*Shigella* sp.		
*Staphylococcus aureus*		
*Vibrio* sp.		
*Yersinia enterocolitica*		

Based on the inherent function of the pathogens, foodborne diseases are classified into “intoxication,” “toxicoinfection,” and “infection.” Intoxication occurs when water or a food product contaminated by pathogenic toxin is ingested. The symptoms of this class appear very quickly. *Staphylococcus aureus*, *Clostridium botulinum*, and *Bacillus cereus* are the most important pathogens cause food intoxication. “Toxicoinfection” results when an ingested pathogen produces a toxin inside the host body. The symptoms of this type are diarrhea and occasional vomiting. *Clostridium perfringens*, *Escherichia coli*, and *Vibrio cholera* are the examples of this class. “Foodborne infection” occurs when an invasive pathogen is ingested. *Salmonella enterica*, *Campylobacter jejuni*, *Escherichia coli* (*E. coli* O157:H7), *Shigella* sp., *Yersinia enterocolitica*, and *Listeria monocytogenes* are the most important bacteria involved in the foodborne infections (Bhunia, 2018).

*Campylobacter* sp. mostly associated with raw or undercooked poultry while *Salmonella* sp. mostly found in meat, poultry, and eggs. *Shigella* sp., and *Escherichia coli* mostly found in meat and unpasteurized milk. *Clostridium botulinum* often found in improperly home-canned foods. *Clostridium perfringens*, *Yersinia*, *Vibrio* sp., *Staphylococcus aureus*, *Bacillus* sp., and *Listeria* are found in uncooked meats, vegetables, unpasteurized milk, and soft cheese (Safavieh et al., 2015).

*Toxoplasma gondii*, *Cryptosporidium parvum*, *Cyclospora cayetanensis*, *Giardia intestinalis*, *Taenia solium*, *Trichinella spiralis*, and Norovirus, hepatitis A virus, and Rotavirus are the most marked parasites and viruses which cause foodborne diseases (Table 1; Bhunia, 2018).

## COVID-19

COVID-19 is an infectious disease caused by SARS-CoV-2 which is manifested by symptoms ranging from mild influenza to severe pneumonia and acute respiratory distress syndrome (Petrosillo et al., 2020). COVID-19 is an enveloped single-stranded RNA virus that its outbreak had become a pandemic in 2020 (Asselah et al., 2021). The beginning of the SARS-CoV-2 outbreak has been attributed to the seafood market in Wuhan, Hubei, China (Chan et al., 2020). According to the WHO, the main route for COVID-19 transmission is respiratory droplets generated by coughing, sneezing, speaking, and breathing of an infected person (Tang et al., 2020). There is no evidence suggesting food is transmission route for COVID-19. The Food and Drug Administration (FDA) (U.S. Food and Drug Administration [FDA], 2020) and CDC emphasize that the risk of infection by the COVID-19 from food products, and food packaging is thought to be very low. Up to now, no cases of COVID-19 have been reported where infection was thought to have occurred by consuming and touching food or food packaging.

Previous studies on coronaviruses family indicated that these viruses can persist in the environment for a long time and may be transmitted through the package surfaces (Geller et al., 2012). Currently, the proposed route for the possible transmission of SARS-CoV-2 through food is the consumption of infected animal foods or the consumption of cross-contaminated foods (Olaimat et al., 1854).

To date, no studies have investigated the survival of SARS-CoV-2 in various foods and food packaging. Therefore, it is not possible to comment at this time on the potential survival of SARS-CoV-2 in food products.

The COVID-19 virus can live on inanimate objects for 72 h (van Doremalen et al., 2020). If the respiratory secretions of a person with corona are secreted onto a food product, it may become a virus carrier. Contact with these food carriers can cause the virus to enter the respiratory system. Since each food product travels a long distance from farm to fork, and along this route they may be contaminated with infectious droplets, regular hand washing and the proper use of sanitizers, and disinfectants are recommended (Olaimat et al., 1854). Besides, the use of online food delivery is advised to create a physical distance between customers and sales staff (U.S. Food and Drug Administration [FDA], 2020). It is also recommended to use cooked foods because coronaviruses do not survive in high temperatures. Raw foods should be washed first and then frozen; because the virus can survive up to 2 years at freezing temperatures (Olaimat et al., 1854).

Food nanopackaging can be a promising approach to maintaining food health against SARS-CoV-2. However, the number of studies that have been done on this subject can be summarized as one where it reported that coatings or films containing copper, silver and zinc NPs have the ability to fight the virus (Sportelli and Izzi, 2020). Antiviral potency of NPs is an open field of research that needs to be fully addressed.

## Antimicrobial Nanopackaging

### Types of Antimicrobial Nanopackaging

**FIGURE 1:**
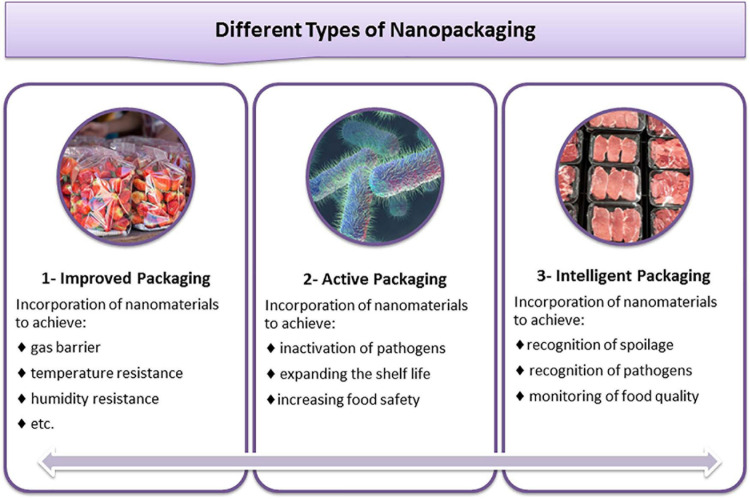
Classification of food nanopackaging and their application for food safety.

Nanopackaging can be divided into three main categories (Figure 1); (i) Improved packaging: These packages contain NPs and are resistant to temperature and humidity; (ii) Active packaging: Packages containing preservatives such as inorganic NPs that can interact directly with food and provide antimicrobial properties; (iii) Intelligent/smart packaging: Packages that are designed for sensing biochemical or microbial changes and specific pathogen developing in the food.

The basic goal of improved packaging is to increase the mechanical and physical properties of the packages. Various nanocomposites are manufactured for various food products such as beverages and oils to reduce the oxygen and carbon dioxide permeation for up to 80–90%. One of the most popular NPs used in improved packaging is nanoclay. Nanoclay incorporated into the polymer can prevent the penetration of oxygen up to 50% and water vapor up to 90% (Kim and Cha, 2014). Antimicrobial properties are not part of the properties of improved packaging.

The development of nanomaterials leads to manufacturing of antimicrobial active packaging that is capable to preserve foods and extending their shelf life. Since the main focus of this study is on antimicrobial nanopackages, we review the active packages in more detail.

### Active Packaging

Food spoilage and foodborne diseases represent a critical health problem. In addition, it will incur a lot of economic and medical costs. The development of new nanotechnologies and nanomaterials for food safety enhancement is required (Mustafa and Andreescu, 2020). Active NPs are nanoscaled materials with intrinsic preservatives antimicrobial and/or antioxidant properties that can be able to exert their activity or releasing the active functions. An active package can release antimicrobials or antioxidants into the package or absorb oxygen or water vapor from inside the package. Incorporation of active materials such as antimicrobial agents and preservatives to conventional non-degradable or biodegradable packaging polymer creates active nanocomposites that are used for food products packaging with the aim of increasing the quality and shelf life of storage food. In addition to increasing microbial safety, active nanopackages can regulate moisture, absorb ethylene, carbon dioxide, oxygen, and water vapor, and act as thermal insulators (Drago et al., 2020). Among the NPs used in the packaging industry, metals [Silver (Ag), Copper (Cu), Gold (Au)], and metal oxides [Titanium dioxide (TiO_2_), Silicon oxide (SiO_2_), Zinc oxide (ZnO)] are the most widely used NPs in active packaging applications (Bikiaris and Triantafyllidis, 2013). Table 2 lists some of the most important articles about active nanopackaging published between 2018 and February 2021.

**TABLE 2:** Some of the most important nanopacking articles from 2018 to February 2021.

**Nanocomposite**	Nanoparticle concentration (method)*	**Tested foodborne pathogens**	Types of food**	**Effect**	**References**
Gelatin/Cellulose nanofibers/ZnONPs/ and or Selenium NPs	Different concentrations (Casting method)	*E. coli*, *L. monocytogenes*, *P. fluorescens*, *S. aureus*	–	A stronger antibacterial effect of ZnONPs compared with SeNPs. The bacterial susceptibility to the antibacterial films was as follows: *L. monocytogenes* > *E. coli* > *S. aureus* > *P. fluorescens*	Ahmadi et al., 2021
Plasticized polylactide/Polyethylene glycol/Ag-Cu NPs/Cinnamon essential oil	4% Ag-Cu NPs with 50% Cinnamon essential oil (Compression-molding method)	*C. jejuni*, *L. monocytogenes, S. Typhimurium*	Chicken meat	Maximum antibacterial action during 21 days at the refrigerated condition	Ahmed et al., 2018
Low-density polyethylene/AgNPs/TiO_2_NPs	0, 1, 3, and 5% of AgNPs (Melt mixing and sol–gel methods)	*A. niger*, *C. albicans*, *E. coli*, *S. aureus*	Pikeperch filets	Effectiveness against all examined bacteria in 3% of AgNPs	Barani et al., 2018
Low-density polyethylene/AgNPs	1.50, 3.75, 7.50, 15.00, 30.00, 60.00, and 75.00 μg/ml of AgNPs	*Apple-isolated Penicillium expansum, E. coli, Enterococcus faecalis, Salmonella enterica* subsp. enterica serovar *Typhimurium, and S. aureus*	–	Antimicrobial effects against all the microorganisms studied, although more notably for fungi and gram-negative bacteria than the gram-positive bacteria	Brito et al., 2020
Cellulose/AgNPs	0.005, 0.01, 0.02, 0.04, and 0.08 g of AgNPs (Using N,N-dimethylacetamide as a reducing agent in the presence of PVP-K30)	*E. coli* and *S. aureus*	–	Remarkable antibacterial activities. The sterilization effect of produced film with 0.04 g of AgNPs against both bacteria exceeds 99.9%	Chen et al., 2020
Low-density polyethylene/AgNPs + CuNPs/TiO_2_NPs	0.1, 0.3, 0.5, 1, 3, and 5% of AgNPs + CuNPs (Melt mixing masterbatch method)	*E. coli* and *L. monocytogenes*	Nile Tilapia fish	a film containing 2.5% of AgNPs and 2.5% of CuNPs had the most significant antimicrobial effect on the Nile Tilapia fish	Efatian et al., 2021
Carboxymethyl cellulose/Cellulose nanocrystal-AgNPs	1, 3, 5, and 7% of AgNPs	*E. coli* and *S. aureus*	Strawberries	The best antibacterial activities against the two bacterial strains Better maintenance of strawberries quality compared with unpackaged strawberries and extending the shelf-life of strawberries to 7 days	He et al., 2021
Chitosan/Nigella sativa extract-AgNPs	0.1% w/v, 0.2% w/v, 0.3% w/v of AgNPs	*B. subtilis*, *E. coli*, *P. aeruginosa*, *S. aureus*	–	A good antibacterial activity against gram-negative bacteria compared to the gram-positive bacteria For both gram-positive and gram-negative bacteria, antibacterial activity significantly influenced by the AgNPs concentration	Kadam et al., 2019
Chitosan-TiO_2_NPs/Red apple pomace extract	10% TiO_2_NPs	*E. coli* and *S. aureus*	–	More effective antimicrobial activities against *S. aureus* than *E. coli*	Lan et al., 2021
Carboxymethyl cellulose/Glycerol/Dioscorea opposita mucilage from Chinese yam/ZnONPs	“CMC to DOM weight ratio” of approximately 1:1, 2.0 g ZnONPs (Casting method)	*E. coli*, *S. aureus*	–	Antibacterial effects against both *E. coli* and *S. aureus*	Li et al., 2021
Low-density polyethylene/AgNPs/TiO_2_NP and Low-density polyethylene/nanoclay/TiO_2_NPs	0.1, 0.3, 0.5, 1, 3, and 5% of AgNPs or nanoclay (Melt mixing and sol–gel methods)	*E. coli*, *S. aureus*	Chicken meat	Greatest antimicrobial effect on gram-positive and gram-negative bacteria for films containing 5% silver and 5% titanium dioxide nanoparticles	Lotfi et al., 2019
Polyvinyl alcohol/Boiled rice starch/AgNPs	6.8 μg/mg AgNPs (Photo-assisted method)	*S. aureus* and *S. typhimurium*	–	Stronger antibacterial activity against *S. typhimurium* than *S. aureus*	Mathew et al., 2019
Cellulose/CuNPs	5, 25, 125, and 250 mM (Simple casting ethanol regeneration method)	*E. coli* and *Bacillus* sp.	–	Remarkable biocidal activity against *E. coli* in 250 mM concentration	Muthulakshmi et al., 2019
Poly (3-hydroxybutyrate-co-3-hydroxyvalerate)/Biogenic SiO_2_NPs	Different concentrations (Solution casting method)	*E. coli* and *S. aureus*	–	Progressively improvement of antibacterial activity of nanocomposites upon increasing SiO_2_NPs concentration	Ojha and Das, 2020
Poly lactic acid/Oligomeric lactic acid/Chitosan-AgNPs	0.5 wt%, 1 wt%, 3 wt%, 5 wt% of AgNPs (Facile and green synthesis method)	*E. coli*, *S. aureus*	–	Antimicrobial activities against the both bacteria. However, the low content of chitosan-AgNPs was not effective against *E. coli* but was against *S. aureus* bacteria	Sonseca et al., 2019
Carrageenan/Laponite on the oxygen plasma surface modified polypropylene film/AgNPs	20 μg/ml AgNPs (Green synthesis method from the *Digitalis purpurea* plant)	*E. coli* and *S. aureus*	–	The excellent antimicrobial activity against the both bacteria	Vishnuvarthanan and Rajeswari, 2019
Chitosan/ZnONPs/Gallic acid	30, 50, and 70 mg of ZnONPs (Facile green method using solution casting technique)	*B. subtilis* and *E. coli*	–	Antimicrobial activities against both bacterial strains	Yadav et al., 2021
Cellulose nanofibril/AgNPs	50–1,000 μg/ml of AgNPs (Reduction method using NaBH_4_)	*E. coli* O157:H7, *L. monocytogenes*	–	Greater inhibitory effect on the growth of *E. coli* O157:H7 than on *L. monocytogenes*	Yu et al., 2019
Poly(lactic acid)/3-(4′-epoxyethyl-benzyl)-5,5-dimethylhydantoin/SiO_2_NPs	1, 3, 5, 7, and 9% of SiO_2_NPs	*E. coli* and *S. aureus*	–	Strong antibacterial activities against both bacterial strains	Zhao et al., 2020

*The nanocomposite fabrication method referred to it directly by the authors was mentioned in the parentheses.

**In addition to routine microbiological tests, some studies have tested the antibacterial properties of fabricated nanocomposites in the packaging of various foods. The type of food studied is listed in this column.

### Silver Nanoparticles

Nanosilver is the most common NPs used for food packaging because of its high stability and its strong toxicity to a wide range of microorganisms. The mechanism of antimicrobial activity of AgNPs is not fully understood, but the following has been suggested by a number of studies. Silver NPs can release silver ions which can adhere to the cytoplasmic membrane and enhance its permeability. Up taking of free silver ions into bacterial cells inactivate respiratory enzymes and generate reactive oxygen species (ROS) but interrupt the adenosine triphosphate (ATP) synthesis. The generated ROS can cause DNA damage and bacterial death (Yin et al., 2020). Numerous studies have improved the shelf life of different food products by application of the antimicrobial activity of AgNPs. Mahdi et al. (2012) reported that trays coated with silver NPs could extend the minced meat shelf life value to 7 days at refrigerator temperature compared with common packaging. Different cellulose types could carry stable silver NPs formed *in situ* by physical and chemical reduction methods. Hybrid materials produced by ultraviolet light and heat were effective against pathogenic microorganisms (mesophiles as well as lactic acid bacteria) on chicken exudates (Fernández et al., 2009). The chitosan silver oxide nanocomposite film showed excellent antibacterial performance against *E. coli*, *B. subtilis*, *P. aeruginosa*, and *S. aureus* microorganisms. This nanocomposite may be used to package foods that are highly sensitive to microbial growth or directly as a surface coating on perishable fruits and vegetables (Tripathi et al., 2011). Different concentrations of AgNPs to reduce total microbial count, mold, and coliform counts in nuts were also reported (Tavakoli et al., 2017). Silver-montmorillonite (Ag-MMT) NPs have also been considered to increase the fresh-cut carrots shelf life up to more than 2 months (Costa et al., 2012). Several studies have investigated the antimicrobial effects of nanosilver in combination with low density polyethylene (LDPE). Becaro et al. (2015) have reported the antibacterial effect of films containing silver NPs embedded in silica or titanium dioxide and were mixed with LDPE. Their films showed antimicrobial properties against *S. aureus* and *E. coli*, presenting better activity against *S. aureus* (Becaro et al., 2015). Lower concentrations of AgNPs incorporated in LDPE had antibacterial performance against mesophilic aerobic and coliforms in fresh-cut carrots packaged. Ascorbic acid content of fresh-cut carrots was also maintained (Becaro et al., 2016). A recent study reported that LDPE/AgNP films had a greater effect on gram-negative bacteria and fungi than gram-positive bacteria (Brito et al., 2020). Additional, antimicrobial properties of nanocomposites of carrageenan/AgNPs/Laponite was demonstrated against the both gram-negative *E. coli* and gram-positive *S. aureus* (Vishnuvarthanan and Rajeswari, 2019). It is well documented that AgNPs have strong antibacterial activity toward gram-negative than gram-positive bacteria (Shankar and Rhim, 2015; Orsuwan et al., 2016). Mathew et al. (2019) observed that both polyvinyl alcohol/AgNPs and polyvinyl alcohol/starch from the boiled red rice/sAgNPs films had stronger antibacterial activity against *S. typhimurium* than *S. aureus*. The difference in the cell wall structure of gram-negative and gram-positive bacteria causes this difference. In fact, the cell wall peptidoglycan layer in gram-positive bacteria makes it difficult for silver NPs to penetrate (Shankar and Rhim, 2017).

### Copper Nanoparticles

Copper NPs are considered to be an antimicrobial agent for medicine and food. The potential biocidal activity of copper is low; however, copper NPs are preferred to silver NPs because of lower cost, easier mixing with polymers, and relatively more physicochemical characteristics.

Copper shows an excellent antimicrobial activity against a wide range of microorganisms. Three different methods including disc diffusion test, minimum inhibitory concentration (MIC) and minimum bactericidal concentration (MBC) have been used by Ruparelia et al. (2008) to study the effects of CuNPs against *E. coli*. This study showed that copper NPs have great promise as antimicrobial agent against *E. coli*, *B. subtilis*, and *S. aureus*. Càrdenas et al. (2009) tested the antimicrobial activity of colloidal CuNPs/chitosan composite film against *S. aureus* and *Salmonella enterica serovar Typhimurium*. They showed the composite film was effective in alteration of cell wall and reduction of microbial concentration in the liquid culture for both bacteria tested (Càrdenas et al., 2009). Copper NPs synthesized using different types of copper salts had antimicrobial activity against *L. monocytogenes* as a gram-positive, and *E. coli* as a gram-negative foodborne pathogens (Shankar and Rhim, 2014). The antifungal activity as well as the antimicrobial activity against *Saccharomyces cerevisiae* has been illustrated in cellulose films containing copper oxide NPs (Llorens et al., 2012). Regardless of the type of microorganism in CuNPs bioactivity experiments, Cu-nano-antimicrobials proved to kill or inhibit the microorganism growth. Laser-generated CuNPs embedded in a biodegradable polymer matrix (polylactic acid) possess good antibacterial activity against *Pseudomonas* sp. (Longano et al., 2012). Polylactic acid films activated by CuNPs showed good antibacterial activity for fresh dairy products (Conte et al., 2013).

### Gold Nanoparticles

Gold NPs have a great antimicrobial activity against several ranges of microorganisms depend upon their size and shape (Lima et al., 2013). Therefore, the biofilms containing gold NPs are very promising to be used as active food packaging for the extension of the food shelf life. Lima et al. (2013) showed that 5 nm AuNPs eliminated 90–95% of *E. coli* and *Salmonella typhi* colonies at short times. Active biofilms of quinoa (Chenopodium quinoa, W.) starch exhibited strong antibacterial activity against *E. coli* and *S. aureus* foodborne pathogens with inhibition percentages of 99 and 98%, respectively (Pagno et al., 2015). Gold NPs probably affect respiratory-chain enzymes which leading to cell death (Zawrah and Abd El-Moez, 2011). AuNPs in combination with bacteriocin had increased antimicrobial activity against four food spoiling organism of *Micrococcus luteus*, *Bacillus cereus*, *S. aureus*, and *E. coli* (Thirumurugan et al., 2013).

### Titanium Dioxide Nanoparticles

Titanium dioxide is a photocatalytic agent used to inactivate a wide range of microorganisms. It is probably able to kill many microorganisms due to its strong oxidizing power and the production of free hydroxyl radicals in near-UV light (Chorianopoulos et al., 2011). The photocatalytic activity of TiO_2_ has been used to purify contaminated water (Chaleshtori et al., 2009). Bodaghi et al. (2013) prepared a TiO_2_ nanocomposite thin film by the extrusion method. They observed that when the film was exposed to UVA light, *Pseudomonas* sp., *R. mucilaginosa* and mesophilic bacteria were inactivated in saline and pear solution (Bodaghi et al., 2013). The antimicrobial activity of the TiO_2_ nanoparticle-coated films has been found at various concentrations (0–0.11 g/100 ml organic solvent) under fluorescent and ultraviolet light (Othman et al., 2014). TiO_2_NPs in gelatin-based films also showed excellent antimicrobial activity against *S. aureus* and *E. coli* (Nassiri and MohammadiNafchi, 2013). According to the Gumiero et al. (2013) study, the high-density polyethylene + CaCO_3_ + TiO_2_ composite matrix was able to provide a greater maintenance of the original cheese structure due to the inhibition of lactic acid bacteria and coliforms. Compared to the TiO_2_ nanoparticle-incorporated film, a hydrothermally synthesized sodium alginate film containing functional Au-TiO_2_ nanocomposites improved the antimicrobial activity by 60 and 50% against *S. aureus* and *E. coli*, respectively (Tang et al., 2018). TiO_2_-ZnO-MgO mixed oxide nanomaterials is another type of TiO_2_ nano-alloy that has shown good antibacterial properties against *E. coli*, *Salmonella paratyphi*, *S. aureus*, and *L. monocytogenes* (Anaya-Esparza et al., 2019). Ansari et al. (2020) studied the synthesis of electrospun TiO_2_ nanofibers, and showed TiO_2_ nanofibers were more active against gram-negative *P. aeruginosa* cells than gram-positive *S. aureus*. They also demonstrated that TiO_2_ nanofibers inhibited biofilm formation of methicillin-resistant *S. aureus* and *P. aeruginosa* in a dose-dependent manner (Ansari et al., 2020). In a recent study, nanosized TiO_2_ and red apple pomace utilized as a potential extraction source to develop an intelligent chitosan-based film for packaging of the freshness of salmon filets. It was showed that this film had a synergistic enhancement of the antimicrobial activity as well as antioxidant property and pH responsive color-changing indicator (Lan et al., 2021).

### Silicon Dioxide Nanoparticles

Silicon dioxide (SiO_2_, silica) NPs possess several advantages such as high strength, thermal stability, high abundance, and low cost used in active polymer-inorganic composite materials (Hou et al., 2019). Recently, the antimicrobial activities of the gellan gum-sodium carboxymethyl cellulose (GC)-SiO_2_ and GC-SiO_2_-octadecyldimethyl-(3-trimethoxysilylpropyl)- ammonium chloride (ODDMAC) nanocomposites were tested against *S. aureus*, *Bacillus cereus*, *Cronobacter sakazakii*, *Salmonella enterica*, *Salmonella typhimurium*, and *E. coli*. The results indicate that the GC-SiO_2_-ODDMAC film had a broad spectrum of antimicrobial activities for both gram-positive and gram-negative pathogens (Rukmanikrishnan et al., 2020). In another study, the nano-SiOx/chitosan complex coating film was applied for improving the tomatoes shelf life and preservation of tomatoes quality. The developed nano film slowed moisture loss, gas exchange, and respiration rates. It also limited bacterial growth, and prevented the increase of malondialdehyde and total polyphenol content. The novel nano-SiOx/chitosan complex film has been proposed for packaging the postharvested tomatoes (Zhu et al., 2019).

### Zinc Oxide Nanoparticles

Zinc oxide is one of the generally recognized as safe (GRAS) material listed by the FDA (Espitia et al., 2012). Recently, a chitosan and zinc oxide NPs loaded gallic-acid films has been proposed for active food packaging for black grape, apple, mango fruits, tomato etc. (Yadav et al., 2021). For extending the shelf life of guava fruits, Kalia et al. (2021) fabricated a chitosan-based nanocomposite film containing CuO and ZnO NPs synthesized from the nettle leaf extract. The antioxidant and antimicrobial activity of biologically synthesized NPs was in order of CuONPs > ZnONPs > nettle extract (Kalia et al., 2021). Foodborne pathogens including *E. coli* O157:H7 can easily grow in white brined cheese. The chitosan and chitosan-ZnONPs coating reduced the initial numbers of *E. coli* O157:H7 in white brined cheese (Al-Nabulsi et al., 2020). Further, functionalized absorbing pads containing ZnONPs have also been successfully applied for controlling *C. jejuni* in raw chicken meat, where the reduced *C. jejuni* count to an undetectable level by immobilized ZnONPs was reported (Hakeem et al., 2020).

### Intelligent Packaging

Intelligent food packaging is a packaging structure that utilize the internal molecules or external conditions of the packed food as information to warn of any changes in the environment of the packages (Drago et al., 2020). Microbiological and chemical tests are regularly carried out on food products, but there is not adequate control after delivery to the supermarket. This unfilled space can be charged with intelligent packaging (Ghaani et al., 2016). Intelligent packaging can be used as a detection method for recognition of spoilage and pathogenic microorganisms. The intelligent packaging can provide information about both the food product status and the environment surrounding it (Vanderroost et al., 2014). They can be equipped with nanosensors classified as freshness indicators, time-temperature indicators, moisture indicators, optical oxygen sensors, optochemical CO_2_ indicators, toxins indicators, ph contaminants indicators, and spoilage and pathogens indicators (Alfei et al., 2020). Most of these sensors or indicators are based on colorimetric methods. Natural dyes such as anthocyanins, curcumin, chlorophyll, and b-carotene found in many fruits and vegetables as well as synthetic dyes based on azo-compounds can be used as sensors in the packaging materials. Enzymatic processes that are capable to catalyze color chemical reactions are good candidate as sensors in intelligent packaging (Halonen et al., 2020). Currently, the selective interaction between antibody and antigen is one of the explored strategies used for intelligent packaging to identify microorganisms. The methods are based on antibodies conjugated with NPs, such as quantum dots (QDs). QDs are nanomaterials made from inorganic semiconductors and possess specific intrinsic characteristics. QDs are a promising class of fluorescent labels for biological detection because they can absorb wide and continuous wavelengths spectra and produce narrow fluorescence emission spectra depending on their size and composition (Valizadeh et al., 2012). QDs conjugated with antibodies are mainly employed due to their specific characteristics for detection of bacteria (Mihindukulasuriya and Lim, 2014). Mohamadi et al. (2017) presented a method to detect distribution of *E. coli* labeled with CdSe-QDs both on an agar nutrient and ground fish substrates. They showed that *E. coli* growth on food products can be easily monitored by CdSe-QDs (Mohamadi et al., 2017). Wang et al. (2011) developed a multiplex immunoassay by integrating magnetic nanobeads (MNBs) for immunoseparation with QDs as fluorescent labels for detection of *Salmonella Typhimurium*, *E. coli* O157:H7, and *L. monocytogenes*, in food products. Their presented multiplex immunoassay method detected simultaneously all three pathogens at levels of 20–50 CFU/ml or lower in food samples (Wang et al., 2011). Recently, a peptide-mediated immunomagnetic separation technique and an immunofluorescence quantum dot technique have been presented for detection of *E. coli* O157:H7, *S. aureus*, and *V. parahaemolyticus*. The method is able to detect three kinds of foodborne pathogens at the same time (Wang et al., 2020). Researchers have recently developed a bacteria-detecting device to detect *L. monocytogenes* in opened food packages. These devices use strips on which gold or palladium nanoparticle-labeled antibodies detect the presence of bacteria (Yousefi et al., 2019).

From the above reports, it can be concluded that the development of new nanomaterials and devices are potential options for producing intelligent labels that can detect pathogens and toxins in food matrix. More future studies need to be done to establish this technique in food antimicrobial nanopackaging application.

### Toxicological Aspects

Comprehensive information regarding the interface between NPs and the human body, particularly in relation to possible NPs hazards to human health must be obtained. NPs incorporated to the nano-packed food products may enter into the body through inhalation, ingestion or cutaneous exposure (Maisanaba et al., 2015). NPs are not soluble in biological fluids, so if they enter the bloodstream, they accumulate in organelles. A very few studies have investigated possible toxicity of NPs combined with food packages. A migration of low molecular mass constituents of nanopackaging to food products is a matter of high concern, both for researchers and consumers. The migration of NPs to food matrix mainly relies on the concentration and particle size of them and composition of food. The migration of metal NPs into the food matrix depends on temperature and acidity (Huang et al., 2015). Few studies points out toward the genotoxicity and carcinogenicity of NPs. Aschberger et al. (2011) observed that toxicity of nanosilver and nano TiO2 can be present in high doses. ZnONPs even at low concentrations may possess a genotoxic potential in human epidermal cells which could be mediated through lipid peroxidation and oxidative stress (Sharma et al., 2009). NPs increase surface to volume ratio which leads to more interaction with biomolecules and create adverse biological responses. It is reported that cationic NPs may be quite toxic than neutral or anionic ones due to their high affinity toward the negatively charged biomolecules (Love et al., 2012). NPs may have adverse effects on circulation, especially affecting microcirculation. If NPs enter the circulatory system, they may affect the blood vessel lining or function and promote blood clot formation or may be associated with cardiovascular effects (Dimitrijevic et al., 2015).

In several *in vitro* studies, genotoxic, cytotoxic, and even carcinogenic aspects of AgNPs were evaluated. It has been observed that the exposure of normal human lung fibroblast cells (IMR-90) and human glioblastoma cells (U251) to 6–20 nm AgNPs disrupted the mitochondrial respiratory chain leading to ROS production and interruption of ATP synthesis. It also induced DNA damage and G2/M phase cell cycle arrest (AshaRani et al., 2009). Proliferating human intestinal cells (Caco-2 cell line) exposure to peptide-coated silver NPs induced ROS production and decreasing adherence capacity (Böhmert et al., 2012). It has been reported that AgNPs were internalized into the cytoplasm of Caco-2 cells and depolarized the mitochondrial membrane potential. In addition, AgNPs depleted the total intracellular glutathione level, induced the activation of Nrf2 (a stress-responsive gene), and increased the expression of heme oxygenase-1 (HO-1) (Aueviriyavit et al., 2014). It concluded that AgNPs induced acute cytotoxicity and cellular responses via the activation of Nrf2/HO-1 signaling pathway in Caco-2 cells (Aueviriyavit et al., 2014). In another study, Miranda et al. (2017) investigated the toxicological interactions of AgNPs (size = 1–2 nm; zeta potential = –23 mV) in human hepatoma HepG2 cells. The co-exposure to AgNPs and metals potentiated cell death mainly by necrosis (Miranda et al., 2017). The synthesized green silver NPs may induce cytotoxicity and cause DNA damage and apoptosis. Bin-Jumah et al. (2020) showed that higher concentrations of green AgNPs reduced cell viability. They also demonstrated following a higher concentration exposure of green AgNPs, count of apoptotic and necrotic cells was increased. These kinds of AgNPs induced more ROS in the HuH-7 cells than in the CHANG cells (Bin-Jumah et al., 2020). Recently, it was reported that AgNPs altered morphology of freshly isolated circulating human peripheral blood mononuclear cells (hPBMC). They induced apoptosis and cell death in a dose- and time-dependent manner (Vuković et al., 2020).

CuNPs have been associated with cytotoxicity mediated through oxidative stress-dependent pathways. Recently, Fahmy et al. (2020) have reported that Cu/CuO NPs suppressed proliferation and viability of normal and carcinoma lung cells. Treatment of both cell types with Cu/CuO NPs resulted in the generation of a state of oxidative stress (Fahmy et al., 2020). In another study, the toxic potentials of CuONPs were evaluated in the two types of human cell lines (HepG2 hepatocarcinoma and Caco-2 colorectal adenocarcinoma). CuONPs were found to cause cytotoxicity, genotoxicity, and oxidative and apoptotic effects in both cell lines (Abudayyak et al., 2020).

AuNPs cytotoxicity was also evaluated in human erythrocytes, murine fibroblasts (NIH3T3), human cervix carcinoma cells (HeLa), and melanoma cells (B16F10) recently. It seems that the physicochemical properties and concentration of AuNPs and also cell type were limiting factors for the cytotoxic effect of AuNPs (Vechia et al., 2020). It has also been found that greatest concentrations of Ag and Ag-Au bimetallic NPs were toxic to both H4IIE-luc (rat hepatoma) and HuTu-80 (human intestinal) cells but AuNPs were not toxic alone. This study suggests that the toxic potency of Ag-Au bimetallic NPs is greater than AuNPs (Botha et al., 2019). However, Enea et al. (2020) showed that the 13 nm AuNPs are toxic to a cell line derived from normal human kidney (HK-2).

According to the studies using TiO_2_ anatase NPs, TiO_2_NPs are cytotoxic or genotoxic. When inhalation is a major rout for entrance of nano TiO_2_ into the human body, the most important adverse effect of TiO_2_NPs in experimental models is pulmonary inflammatory responses and lung cancers (Shi et al., 2013). As reported by Koeneman et al. (2010), TiO_2_NPs at 10 μg/ml and above, can go through the epithelial lining by transcytosis. TiO_2_ could penetrate into and through the cells without disrupting junctional complexes. Low concentrations of TiO_2_ do not disrupt epithelial integrity and cell death (Koeneman et al., 2010). It seems that low doses of nano TiO_2_ are non-toxic.

According to the study conducted by Tarantini et al. (2015) silica NPs penetrate into the cytoplasm but not the nucleus of the human intestinal Caco-2 cell line and have toxic effects in the cells. They proposed that genotoxic effects of silica NPs are likely to be mediated through oxidative stress rather than a direct interaction with the DNA (Tarantini et al., 2015). It has been reported that silica NPs with 100 μg/ml concentration and 24-h exposure are safe for GES-1 and Caco-2 cells. However, at a higher concentration and longer exposure period, they arrest cell cycle and inhibit the cell growth (Yang et al., 2014). In contrast to the above reports, Schübbe et al. (2012) study showed neither cytotoxic nor genotoxic effects were detected for either 32 or 83 nm fluorescent silica NPs.

A significant inhibition of Caco-2 cell viability exposed to ZnONPs (3, 6, and 12 mM) for a 24-h exposure has been reported. Alteration in cell shape, abnormal nuclear structure, membrane blebbing, and cytoplasmic deterioration were the observed changes (Mao et al., 2016). In another study, treatment of rats with 50 and 100 mg/kg ZnONPs induced significantly intestinal injury, while treatment with 5 mg/kg ZnONPs normalized intestinal functions and structure. The authors concluded that ZnONPs have synergistic effects on liver enzyme, oxidative stress, apoptosis, inflammation, morphological changes and cell toxicity (Abbasi-Oshaghi et al., 2018). Caco-2 cells could be protected from ZnONP exposure by myricetin through modulating the apoptosis-ER stress pathway. In fact, co-exposure to myricetin and ZnONPs led to a significantly reduced ratio of cleaved caspase-3/pro-caspase-3 compared with the exposure to ZnONPs (Wu et al., 2019).

From the above reports, it can be concluded that there is no definite answer to the question of whether NPs are toxic. Studies have reported that NPs characteristics including shape, size, size distribution, structure, composition, surface functionality, porosity, surface area, surface charge, agglomeration, concentration, and solubility are all important in the toxicity of NPs (McCracken et al., 2016). For evaluation of NPs toxicity, biological and pathological effects of NPs should be determined by a number of variables, including NPs physiochemical properties, concentration, dose, exposure route, and duration. It seems that the size of NPs, the dose and the exposure time have a great effect on their toxicity. Current data are based on *in vitro* cell culture studies and/or *in vivo* animal model experiments. The potential and power of these models for predicting the NPs toxicity in humans is debated. Great care must be taken in using existing models to investigate and understand the biological and pathophysiological mechanisms of NPs toxicity. Full risk evaluations for various routes of exposure to different types of NPs are required.

## Conclusion

Consumption of foods contaminated with foodborne microorganisms leads to foodborne diseases. The need to avoid foodborne pathogens contamination has led to the development of new preservative methods and innovative packaging. Antimicrobial packaging can play an important role in reducing the risk of pathogen contamination, and improving the quality and shelf life of foods. This review underlines the capability of active and intelligent packages as antimicrobial agents against foodborne pathogens. Large numbers of scientific studies have demonstrated that active and intelligent packaging has many advantages in terms of food safety. Nanomaterials can provide new antimicrobials with wide spectrum of activity and improve durability and shelf life of food products. It is predicted that nanotechnology and nanopackaging could become part of the food industry and change the packaging process and fabrication in the coming years forward. However, a gap still exists, where the toxicity of NPs and their safe applications is controversial. The safety issues and environmental impact should be concerned before releasing the NPs to the market in order to guarantee the human’s health. Given the many benefits of nanopackaging in preserving foods, increasing the shelf-life, and preventing humans from foodborne illnesses, more focused research in the area of optimization of variables caused NPs toxicity must be carried out. A successful participation and collaboration between research activities and industry will promote the antimicrobial nanopackaging technologies.

## Author Contributions

MA assisted in compilation and proper editing of this review. All authors contributed to the article and approved the submitted version.

## Conflict of Interest

The authors declare that the research was conducted in the absence of any commercial or financial relationships that could be construed as a potential conflict of interest.
